# Serum Cardiac Troponin T Levels as a Therapy Response Marker in Tofersen‐Treated ALS

**DOI:** 10.1002/mus.28453

**Published:** 2025-06-09

**Authors:** Sarah Bernsen, Rachel Fabian, Yasemin Koc, Peggy Schumann, Peter Körtvélyessy, Sergio Castro‐Gomez, Thomas Meyer, Patrick Weydt

**Affiliations:** ^1^ Department of Neuromuscular Diseases, Center for Neurology University Hospital Bonn Bonn Germany; ^2^ Deutsches Zentrum für Neurodegenerative Erkrankungen, Research Site Bonn Bonn Germany; ^3^ Department of Neurology, Center for ALS and Other Motor Neuron Disorders, Charité—Universitätsmedizin Berlin, Corporate Member of Freie Universität Berlin Humboldt‐Universität Zu Berlin and Berlin Institute of Health Berlin Germany; ^4^ Department of Parkinson, Sleep and Movement Disorders, Center for Neurology University Hospital Bonn Bonn Germany; ^5^ Ambulanzpartner Soziotechnologie APST GmbH Berlin Germany; ^6^ Deutsches Zentrum für Neurodegenerative Erkrankungen, Research Site Magdeburg Magdeburg Germany; ^7^ Institute of Physiology II, University Hospital Bonn Bonn Germany

**Keywords:** amyotrophic lateral sclerosis, biomarker, SOD1, tofersen, troponin

## Abstract

**Introduction/Aims:**

Cardiac troponin T (cTnT) levels are elevated in the majority of persons with amyotrophic lateral sclerosis (ALS) and increase over time. Neurofilament light chain (NfL) is an established therapy response biomarker in ALS as superoxide dismutase1 (*SOD1*)‐ALS patients treated with the antisense oligonucleotide tofersen show a decrease in NfL. In this study, we assess cTnT levels in *SOD1*‐ALS at baseline and during tofersen treatment.

**Methods:**

cTnT was analyzed at baseline and during tofersen treatment in 23 *SOD1*‐ALS patients at two specialized ALS centers in Germany and compared to a control cohort of 74 ALS patients without *SOD1* variants.

**Results:**

cTnT levels increased in the control ALS cohort over time (*p* < 0.0001) but not in the tofersen group (*p* = 0.36). Creatine kinase (CK) and CK‐MB levels did not show significant changes over time. The median monthly increase of cTnT was 0.045 points (IQR 0.02–0.08) in the control ALS cohort and 0.01 points (IQR −0.01–0.03) in the tofersen group (*p* = 0.0013). A significantly lower fold change in cTnT levels was observed in the tofersen‐treated cohort (median 1.2; IQR 0.77–1.59) relative to the control group (median 1.89; IQR 1.35–2.75) (*p* = 0.0003). Nine (39%) patients treated with tofersen experienced a reduction in cTnT levels.

**Discussion:**

In this study, we describe a response signal of cTnT to tofersen treatment, which supports the value of cTnT as an independent biomarker in ALS. These results contribute to the notion that cTnT may provide additional value as a progression and treatment response biomarker in ALS complementary to NfL and warrant further investigation.

## Graphical abstract

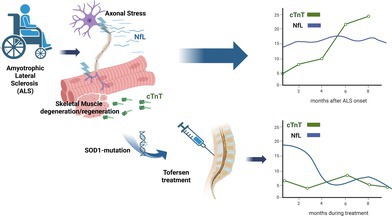

**Abbreviations**
ALS: amyotrophic lateral sclerosis
ALSFRS‐R: Amyotrophic Lateral Sclerosis Functional Rating Scale‐Revised
BMI: body mass index
CK: creatine kinase
Con: control
CSF: cerebrospinal fluid
cTnT: cardiac Troponin T
IQR: interquartile range
Kg: kilogram
L: liter
m: meter
NfL: neurofilament light chain
Ng: nanogram
N/n: Number
PR: progression rate
SEM: standard error of the mean
SOD1: superoxide dismutase 1
Tof: tofersen
U: unit

## 1 Introduction/Aims

Recently the antisense oligonucleotide tofersen was approved as a disease‐modifying drug for the small subset of patients with amyotrophic lateral sclerosis (ALS) carrying a pathogenic superoxide dismutase 1 (*SOD1*) gene variant [1]. In these patients neurofilament light chain (NfL) levels in serum and cerebrospinal fluid (CSF), markers of neuroaxonal stress, are elevated and drop after treatment initiation [2].

Serum cardiac troponin T (cTnT) levels are chronically elevated in nearly two thirds of the ALS patients [3] and closely correlate with disease severity and progression [4, 5, 6]. Of note, cTnT levels are independent from NfL levels and their response to therapeutic interventions is unknown. Unlike other serum markers such as NfL, CK, or CK‐MB, cTnT levels increase over time [5, 6].

New disease modifying interventions offer the opportunity to investigate the potential of cTnT as a therapy response marker. Here, we analyzed the temporal dynamics of cTnT in ALS‐*SOD1* patients treated with tofersen.

## 2 Methods

### 2.1 Study Design

This study is a retrospective analysis using clinical routine data (Bonn) and data from the “APST‐registry study,” and “NfL‐ALS”‐study in Berlin.

### 2.2 Subject Samples

Tofersen is now standard of care for *SOD1*‐ALS, so placebo‐controlled trials for these patients are no longer ethically feasible. Thus, we analyzed demographic and clinical information, and serum samples of *SOD1*‐ALS patients treated with tofersen for at least six treatment cycles at two German ALS clinics providing tofersen treatment (Bonn, Berlin), and compared them to ALS patients without the *SOD1* variant who were not treated with tofersen.

Data for the tofersen treatment group were collected in the 4‐week treatment intervals between June 2022 and July 2024 in Berlin and Bonn.

Longitudinal data from ALS patients without *SOD1* variants were collected at the Bonn ALS clinic between January 2019 and August 2023 and only included in the analysis if ≥ 3 cTnT measurements over > 4 months were available.

As the laboratory measurements and clinical data in Bonn of the treatment and control groups were part of the routine clinical work‐up and retrospectively analyzed, no formal consent was needed according to our institutional ethics review board (Ethics Board decision letter 324/20, Bonn).

At the ALS clinic Berlin, data of the *SOD1*‐patients (demographics, ALSFRS‐R, clinical characteristics, NfL, cTnT, CK, and CK‐MB) were prospectively obtained via the APST registry and its “NfL‐ALS” substudy. The study protocol for the APST registry and the substudy “NfL‐ALS” were approved by the Medical Ethics Committee of Charité‐Universitätsmedizin Berlin, Germany, under numbers EA2/168/20 and EA1/219/15.

### 2.3 Laboratory Markers

#### 2.3.1 Bonn

All measurements were performed at fully accredited commercial laboratories as described previously [5]; CK and CK‐MB (University Hospital Bonn central laboratory), cardiac cTnT (Labor Volkmann, Karlsruhe), serum NfL (University Medical Center of Ulm, Germany).

#### 2.3.2 Berlin

cTnT, CK, and CK‐MB concentrations were measured at the Labor Berlin—Charité Vivantes GmbH. Serum NfL concentrations were analyzed at the ALS center in Berlin, described previously [2].

### 2.4 Variables

Clinical data included sex, body mass index (BMI), age at symptom onset, age at treatment onset (*SOD1* cohort), or age at first visit (untreated ALS cohort), and disease duration. Symptom onset was defined as the date (in month and year) of the onset of motor functional deficits, as captured by the ALS‐Functional Rating Scale Revised (ALSFRS‐R) [7]. Progression rate (PR) (points of ALSFRS‐R lost per month) was obtained and further classified in patients with slower (< 0.5 ALSFRS‐R/month), intermediate (≥ 0.5 and ≤ 1.0 ALSFRS‐R/month), and faster (> 1.0 ALSFRS‐R/month) progression [8].

### 2.5 Statistical Analysis

Statistical description and analyses were performed using GraphPad Prism 10 (GraphPad, San Diego, California, USA). Biorender (Toronto, ON, Canada) was utilized for figures.

cTnT, NfL, CK, and CK‐MB presented a skewed distribution, so data are displayed as medians, and nonparametric tests were used. Differences in group characteristics and biomarkers were analyzed using the Chi‐square test for categorical variables and the Mann–Whitney U test for nonparametric variables. To analyze changes over time between baseline and last visit, the Wilcoxon matched pairs signed rank test was applied. Because of missing values, cTnT, CK, and CK‐MB samples were analyzed by a mixed‐effects model instead of a repeated‐measures analysis of variance. Data were log‐transformed for the mixed effects analysis to approximate normal distribution. Missing values were not replaced. *p* ≤ 0.05 was considered significant.

## 3 Results

### 3.1 Clinical Characteristics

Clinical and demographic characteristics of the tofersen treated *SOD1*‐ALS patients (*N* = 23) and the control ALS group (*N* = 74) are summarized in Table 1. *SOD1* patients were significantly younger and had a lower PR compared to controls, while ALSFRS‐R scores at baseline did not differ significantly. Slow progressors showed no significant difference in baseline PR or ALSFRS‐R.

**TABLE 1:** Demographic and clinical characteristics of the two cohorts.

	ALS, control	ALS, tofersen treated	*p* value for difference
Number of patients	*n* = 74	*n* = 23	
Female, *n* (%)	25 (34%)	11 (48%)	0.2234
Age at onset, median (IQR), years, (*n*)	61 (56.05–71.53), (74)	51.9 (38.7–60.6), (23)	< 0.0001
Age at baseline, median (IQR), years, (*n*)	62.8 (58.18–73.2), (74)	58.8 (49.8–65), (23)	0.0043
BMI at baseline (kg/m^2^), median (IQR), (*n*)	24.85 (22.4–27.75), (60)	24.49 (22.1–27.55), (22)	0.7064
Type of onset, *n* (%)	Bulbar: 23 (31%)	Bulbar: 1 (4%)	0.0095
	Spinal: 51 (69%)	Spinal: 22 (96%)	
Genetic mutation other than SOD1	14 (19%)	—	
ALSFRS‐R at baseline, median (IQR), (*n*)	36 (32–41), (59)	34 (28.5–41.25), (22)	0.2841
ALSFRS‐R at last visit, median (IQR), (*n*)	24 (18–29), (63)	34 (24–42), (15)	0.0013
Tofersen treatments, median, IQR	—	16 (7–20)	—
Months of follow‐up, median (IQR), (*n*)	12 (9–19.25), (74)	14 (6–22), (23)	0.6808
Onset to baseline, months, median (IQR), (*n*)	14 (7.8–124), (74)	43 (22–80), (23)	< 0.0001
PR (onset to baseline) (median, IQR), (*n*)	0.64 (0.33–1.1), (59)	0.31 (0.15–0.58), (22)	0.0014
PR (onset to last visit) (median, IQR)	0.78 (0.55–1.36), (63)	0.21 (0.11–0.49), (15)	< 0.0001
Slow progressors^a^, *n* (%)	23 (32%).	16 (73%)	
Female, *n* (%)	7 (30%)	7 (44%)	
Age at onset, median (IQR), years, (*n*)	58.4 (55.4–74.1), (23)	51.15 (43.25–60.25), (16)	0.02
Age at baseline, median (IQR), years, (*n*)	61.8 (56.8–73), (23)	59.2 (51.83–66.2), (16)	0.14
ALSFRS‐R at baseline, median (IQR), (*n*)	37 (32–45.5), (21)	35.5 (29–45), (16)	0.18
ALSFRS‐R at last visit, median (IQR), (*n*)	25.5 (18.25–30.25), (18)	34 (29–42), (11)	0.0071
Months of follow‐up, median (IQR), (*n*)	18 (12–28), (23)	14 (6–22), (16)	0.1484
PR (onset to baseline) (median, IQR), (*n*)	0.28 (0.23–0.34), (21)	0.19 (0.11–0.32), (16)	0.1241
PR (onset to last visit) (median, IQR)	0.4 (0.28–0.5), (18)	0.2 (0.1–0.24), (11)	0.0008

Abbreviations: ALS, amyotrophic lateral sclerosis; ALSFRS‐*R*, amyotrophic lateral sclerosis functional rating scale revised; BMI, body mass index; IQR, interquartile eange; N/*n*, number; PR, progression rate; SOD1, superoxiddismutase1.

^a^ PR < 0.5 ALSFRS‐R/month.

### 3.2 Serum Biomarkers

At baseline, the tofersen‐treated cohort presented with higher CK‐MB levels and lower NfL levels compared to the non‐*SOD1* ALS patients. Biomarker details are described in Table 2.

**TABLE 2:** Serum biomarker characteristics of the two cohorts.

	ALS, control	ALS, tofersen treated	*p* value for difference
First cTnT in serum (ng/l) (median, IQR), (*n*)	20.7 (11.58–32.75), (74)	20.8 (11–42), (23)	0.7956
Last cTnT in serum (ng/l) (median, IQR), (*n*)	35.1 (19.65–64.2), (74)	19 (12–57), (23)	0.128
First cTnT in Serum > cut‐off^a^, (*n*)	54 (73%), (74)	15 (65%), (23)	0.4734
First NfL in serum (ng/l) (median, IQR), (*n*)	113.5 (91.25–171), (22)	57.8 (21.47–78), (23)	< 0.0001
Last NfL in serum (ng/l) (median, IQR), (*n*)	135 (70–174), (35)	22.7 (9.6–54.6), (22)	< 0.0001
First CK in serum (U/l) (median, IQR), (*n*)	200 (116.5–347), (61)	208 (119.5–443.5), (21)	0.5388
Last CK in serum (U/l) (median, IQR), (*n*)	122 (77.5–239.5), (65)	190 (117–375), (23)	0.0655
First CK‐MB in serum (U/l) (median, IQR), (*n*)	7.5 (3.8–12.6), (60)	14.9 (8.1–20.4), (20)	0.0013
Last CK‐MB in serum (U/l) (median, IQR), (*n*)	6.3 (4.1–12.5), (64)	12.9 (6.2–17.1), (23)	0.008
Slow progressors^b^			
First cTnT in serum (ng/l) (median, IQR), (*n*)	25.7 (10.3–51.8), (23)	17 (11.5–38.5), (16)	0.6162
Last cTnT in serum (ng/l) (median, IQR), (*n*)	56.9 (14.7–75), (23)	18 (10.5–53.6), (16)	0.0695
First NfL in serum (ng/l) (median, IQR), (*n*)	103.5 (59–141.3), (4)	50.55 (24.09–70.9), (16)	0.064
Last NfL in serum (ng/l) (median, IQR), (*n*)	77 (40–169.5), (12)	22.4 (11.2–39), (15)	0.0006
First CK in serum (U/l) (median, IQR), (*n*)	190 (93.25–271.3), (20)	209 (154–463), (15)	0.1687
Last CK in serum (U/l) (median, IQR), (*n*)	108 (79–183), (19)	201 (123–399), (16)	0.0397
First CK‐MB in serum (U/l) (median, IQR), (*n*)	7.7 (3.8–11.6), (20)	14.9 (9.6–19.9), (14)	0.0050
Last CK‐MB in serum (U/l) (median, IQR), (*n*)	6.2 (3.3–12.95), (18)	12.1 (6.4–14.95), (16)	0.0605

Abbreviations: CK, creatine kinase; cTnT, cardiac Troponin T; IQR, interquartile range; NfL, neurofilament light chain; PR, progression rate.

^a^ cut‐off > 14 ng/L.

^b^ PR < 0.5 ALSFRS‐R/month.

cTnT levels in the control‐cohort increased over time (*p* < 0.0001). In the tofersen‐treated patients, cTnT levels remained unchanged (*p* = 0.36). CK in the tofersen cohort, and CK‐MB levels in both groups (baseline vs. last sampling) did not show a significant change. CK levels showed a significant decrease over time only in the control group (*p* = 0.008) (Figure S1A,B).

The compound monthly growth rate for cTnT was 0.045 points (IQR 0.02–0.08) in the ALS control cohort and 0.01 points (IQR −0.01–0.03) in the tofersen patients (*p* = 0.0013). A similar trend was shown in the slow PR subgroup with 0.03 points (IQR 0.01–0.06) in the control cohort and 0.01 (IQR −0.01–0.03) in the tofersen group (*p* = 0.067).

**FIGURE 1:**
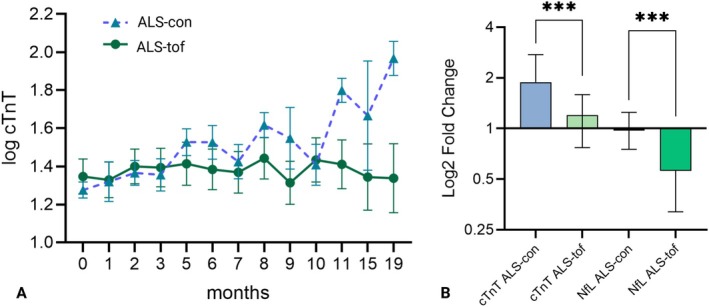
(A) cTnT changes over time (mean, SEM) of SOD1‐ALS patients treated with tofersen (ALS‐tof) and control ALS‐patients (ALS‐con) without tofersen treatment prior to first tofersen administration (ALS‐tof) or first visit (ALS‐con) up to 19 months compared with a mixed linear effects model for repeated measures (*p* = 0,0013). (B) Fold Change of cTnT and NfL from SOD1‐patients treated with tofersen (ALS‐tof) compared with control ALS patients (ALS‐con) without tofersen treatment (cTnT***p = 0.0003, NfL***p = 0.0005). Whiskers show IQR.

Moreover, we measured cTnT repeatedly over time during 19 months. A mixed‐effects model for repeated measures demonstrated a statistically significant interaction of time and treatment for cTnT levels between the two groups (*p* = 0.0013; Figure 1A). This effect remained significant within the slow PR subgroup (*p* = 0.0051) (Figure S2), whereas no significant interactions were observed for CK (*p* = 0.5734) or CK‐MB (*p* = 0.4344) levels.

The fold‐change in cTnT levels of the tofersen‐cohort (median 1.2; IQR 0.77–1.59) was significantly less compared to the control ALS patients (median 1.89; IQR 1.35–2.75) (*p* = 0.0003). Nine patients (39.1%) of the tofersen‐cohort showed a reduction in cTnT levels. This difference remained consistently statistically significant if stratified for the slow PR subgroup (*p* = 0.009). There was no difference in fold change of CK and CK‐MB (data not shown). The fold change in NfL levels decreased in the *SOD1* patients (median 0.56; IQR 0.32–0.85) compared to the control group (median 1.01; IQR 0.75–1.25) (*p* = 0.0005) (Figure 1B).

Due to variability in follow‐up durations across subjects, we normalized the data by calculating the change in biomarker levels (Δ) over the longest available time interval, dividing it by the elapsed time (in days). There was a significant difference between *SOD1*‐ and control ALS patients in terms of Δ cTnT (*p* = 0.0085) (Figure S3) and Δ CK‐MB (*p* = 0.0085), but not Δ CK (*p* = 0.82). Even stratified for the subgroup of the slow progressors, the effect remained statistically significant for Δ cTnT (*p* = 0.003).

## 4 Discussion

In this study, we describe a response signal of serum cTnT levels to tofersen treatment in ALS patients. While cTnT levels increase over time in untreated patients regardless of PR, they stabilize under tofersen therapy. cTnT shows a response to tofersen treatment similar to the previously reported effect on NfL in serum and CSF, thus suggesting cTnT may have use as a complementary biomarker for detecting therapeutic effects in ALS patients. Serum NfL levels were lower in the tofersen‐treated group at baseline reflecting the overall lower PR in this group. This corresponds with the higher CK‐MB baseline levels in this group, as those coincide with slower disease progression [9]. Of note, the absence of the cTnT rise was a feature not only of the entire tofersen group, but also the slow progressing subgroup. This mitigates the concern that the observed cTnT signal lowering is merely an artifact of the mismatch between the two comparator groups, as the comparison of the much more closely matched slow progressing subgroups yielded the same effect.

In line with previous studies, CK and CK‐MB showed no consistent changes over time [9]. The declining CK levels over time in both ALS cohorts may reflect progressive loss of muscle mass. The results of the present study highlight the potential of cTnT as a marker of peripheral involvement in ALS. Interestingly, this peripheral, muscle‐derived biomarker exhibits a dynamic response to a centrally acting therapy. The broad availability, temporal dynamics, and pattern of cTnT distinguish it from NfL, CK, and CK‐MB, and may indicate a potential role in evaluating therapeutic responses.

Although the effectiveness of tofersen treatment in slow progressors has not been formally tested [1, 10], we observed a reduction in NfL levels in this subgroup. This suggests that it may be possible to detect additional biomarker signals of the treatment.

Limitations are the small treatment group and the predominance of *SOD1*‐ALS patients with slow PRs. We have attempted to address this by stratification for the subgroup of slow progressors. A comparison with untreated *SOD1*‐patients is not feasible, as all *SOD1*‐patients in our clinics are on treatment with tofersen.

Our findings support the value of cTnT as an independent biomarker in ALS, delivering information on therapy response and disease activity that likely warrants further investigation in future studies.

## Author Contributions

**Sarah Bernsen:** conceptualization, writing – original draft, methodology, visualization, writing – review and editing, investigation, software, formal analysis, data curation, project administration. **Rachel Fabian:** conceptualization, writing – review and editing, data curation. **Yasemin Koc:** data curation. **Peggy Schumann:** data curation. **Peter Körtvélyessy:** writing – review and editing, conceptualization. **Sergio Castro‐Gomez:** writing – review and editing, visualization, conceptualization. **Thomas Meyer:** writing – review and editing, project administration, supervision, resources, data curation, conceptualization, funding acquisition. **Patrick Weydt:** investigation, conceptualization, funding acquisition, writing – review and editing, project administration, supervision, resources, data curation.

## Ethics Statement

The authors confirm that we have read the Journal's position on issues involved in ethical publication and affirm that this report is consistent with those guidelines.

## Conflicts of Interest

The authors declare no conflicts of interest.

## Supporting information


**Figure S1.** (A) Baseline levels and (B) last visit (log) of cTnT, CK, CK‐MB, and NfL of SOD1‐ALS patients treated with tofersen (ALS‐tof) and control ALS‐patients (ALS‐con) without tofersen treatment. Violin plot represents the actual distribution median (dashed line) and quartiles (dotted line).

**Figure S2.** cTnT changes over time (mean, SEM) of slow progressing SOD1‐ALS patients treated with tofersen (ALS‐tof) and slow progressing control ALS‐patients (ALS‐con) without tofersen treatment prior to first tofersen administration (ALS‐tof) or first visit (ALS‐con) up to 19 months compared with a mixed linear effects model for repeated measures (*p* = 0.0051).

**Figure S3.** Change in time (*Δ* cTnT/days) of cTnT levels of SOD1‐patients with tofersen treatment (ALS‐tof) and control ALS patients (ALS‐con) without tofersen treatment between baseline and last visit. The two groups are compared with Mann–Whitney test (upper asterisks; p** = 0.0085), the level of significance below each group is from Wilcoxon Signed Rank Test on *Δ* cTnT concentrations compared to the theoretical median of no change (p*** < 0.0001). Violin plot represents the actual distribution median (dashed line) and quartiles (dotted line).

## Data Availability

The data that support the findings of this study are available from the corresponding author upon reasonable request.
