# Supplementary figures and images for: Serum Cardiac Troponin T Levels as a Therapy Response Marker in Tofersen‐Treated ALS
Source: Muscle Nerve. 2025 Jun 9;72(3):509–14. doi: 10.1002/mus.28453 (PMC12338008; doi:10.1002/mus.28453)

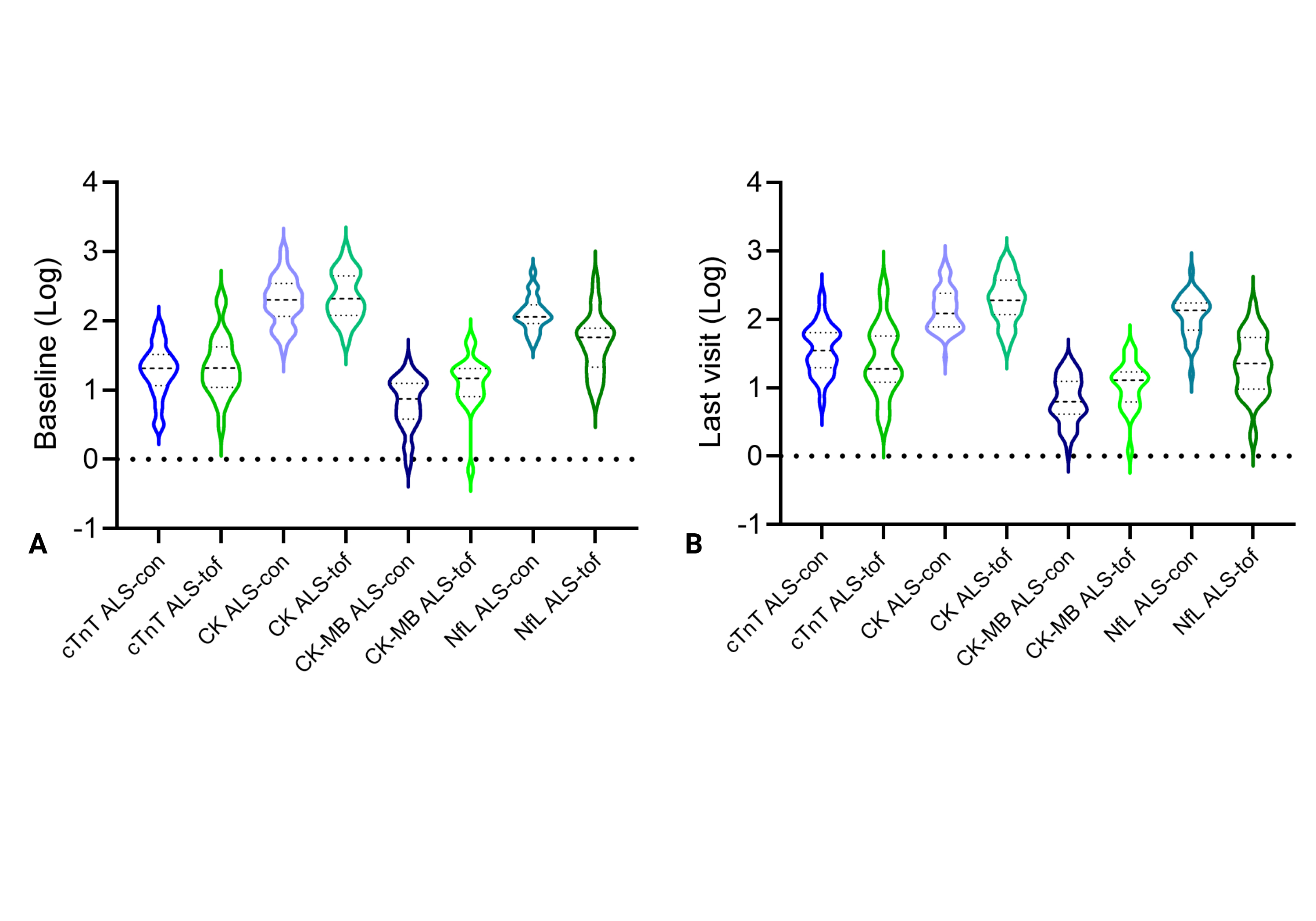

Supplement: Supplementary file 1 — Figure S1. (A) Baseline levels and (B) last visit (log) of cTnT, CK, CK‐MB, and NfL of SOD1‐ALS patients treated with tofersen (ALS‐tof) and control ALS‐patients (ALS‐con) without tofersen treatment. Violin plot represents the actual distribution median (dashed line) and quartiles (dotted line). [file MUS-72-509-s001.jpeg]

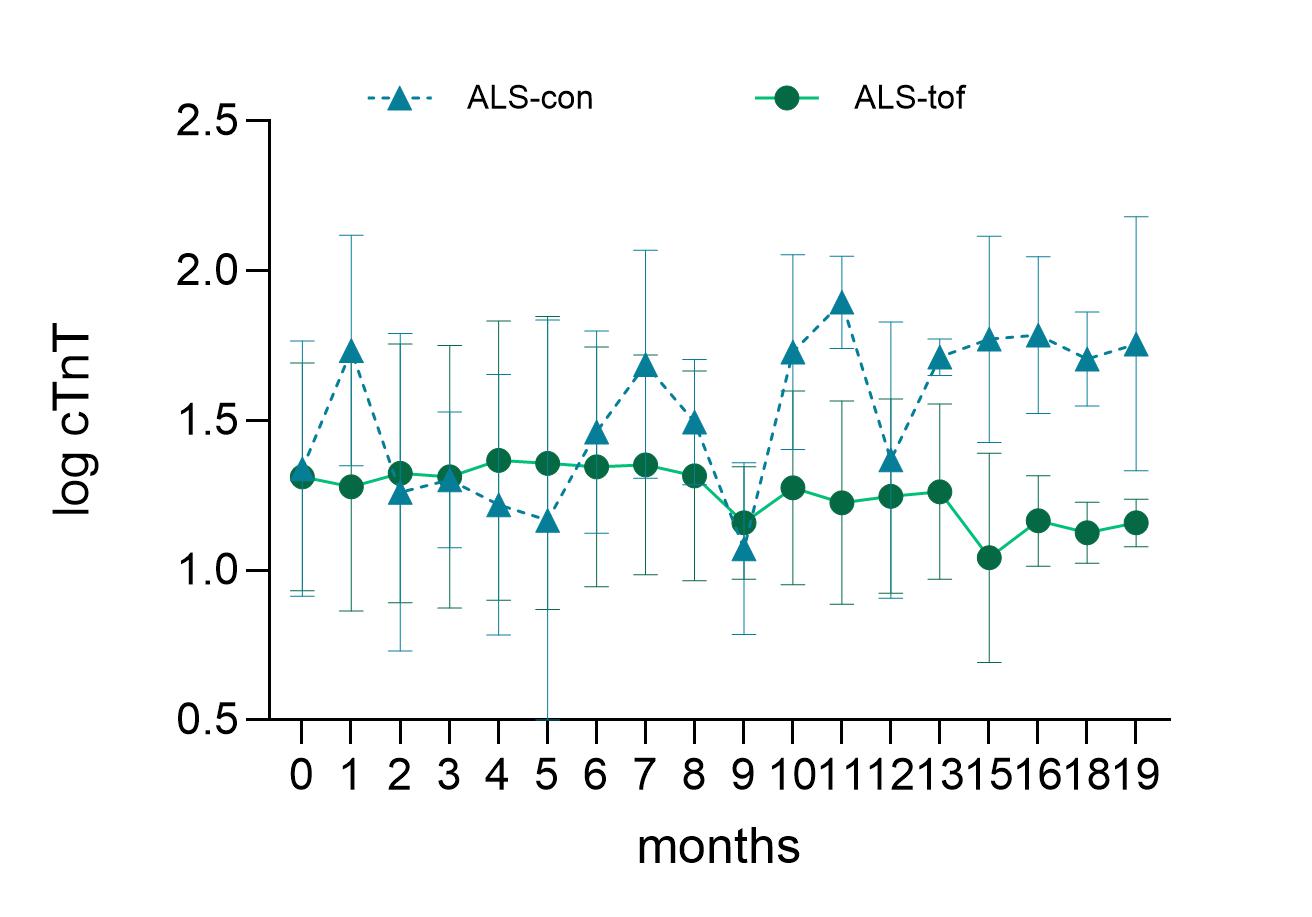

Supplement: Supplementary file 2 — Figure S2. cTnT changes over time (mean, SEM) of slow progressing SOD1‐ALS patients treated with tofersen (ALS‐tof) and slow progressing control ALS‐patients (ALS‐con) without tofersen treatment prior to first tofersen administration (ALS‐tof) or first visit (ALS‐con) up to 19 months compared with a mixed linear effects model for repeated measures (p = 0.0051). [file MUS-72-509-s002.jpg]

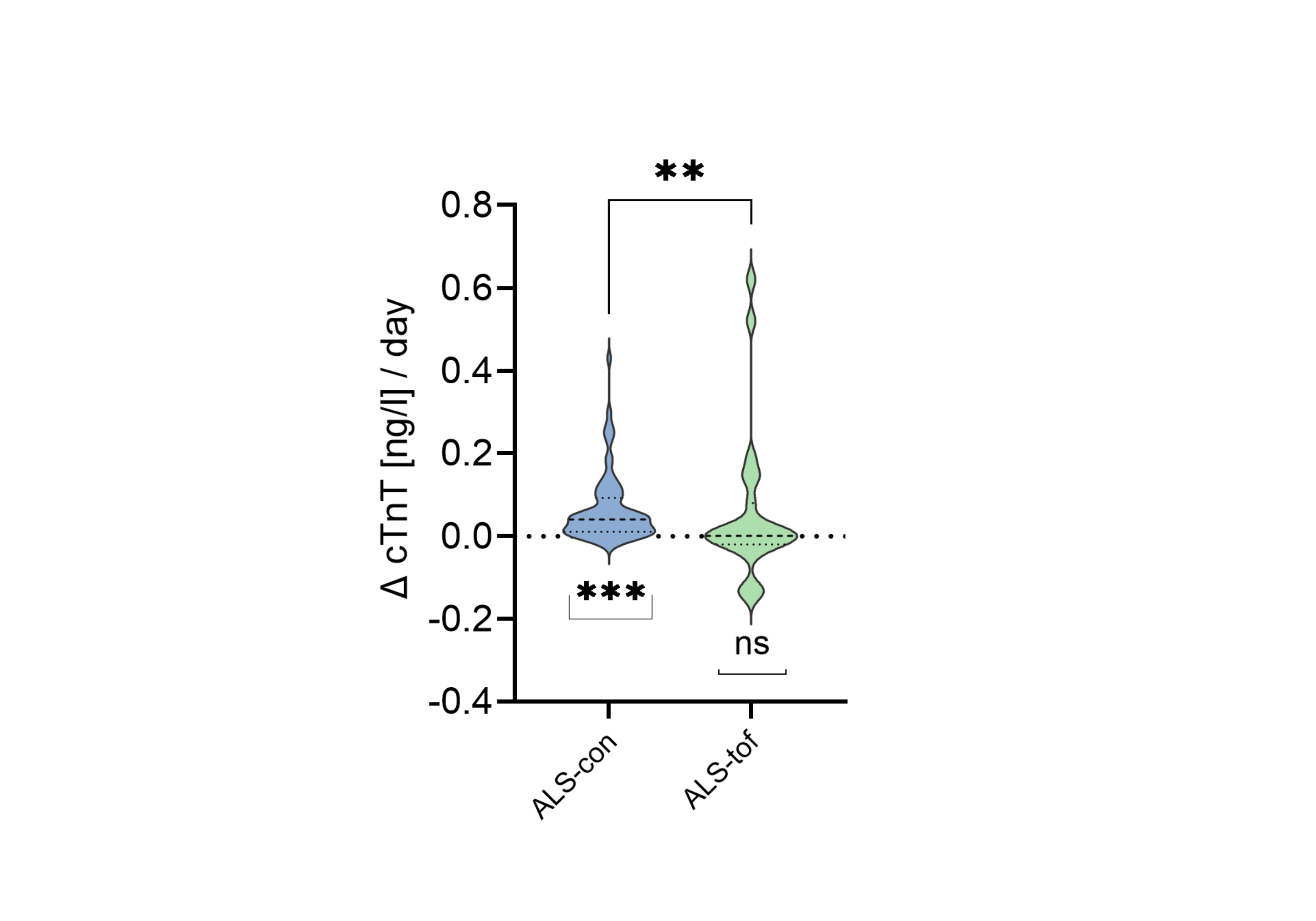

Supplement: Supplementary file 3 — Figure S3. Change in time (Δ cTnT/days) of cTnT levels of SOD1‐patients with tofersen treatment (ALS‐tof) and control ALS patients (ALS‐con) without tofersen treatment between baseline and last visit. The two groups are compared with Mann–Whitney test (upper asterisks; p** = 0.0085), the level of significance below each group is from Wilcoxon Signed Rank Test on Δ cTnT concentrations compared to the theoretical median of no change (p*** < 0.0001). Violin plot represents the actual distribution median (dashed line) and quartiles (dotted line). [file MUS-72-509-s003.jpeg]
